# A Feasibility Evaluation of Discovery Group: Determining the Acceptability and Potential Outcomes of a Patient-led Research Group in a Secure Mental Health Inpatient setting

**DOI:** 10.1192/bjo.2022.170

**Published:** 2022-06-20

**Authors:** Mudassar Arslan, Anne Aboaja, Oluwatosin Atewogboye, Lucia Parry-Newton, Lindsey Wilson

**Affiliations:** Tees, Esk and Wear Valleys NHS Foundation Trust, Middlesbrough, United Kingdom

## Abstract

**Aims:**

Patient and public involvement and engagement (PPIE) is recognised as an essential part of health research. It provides an opportunity for patients to shape health research and acquire research skills, in the inpatient mental health setting, PPIE may have additional value in providing meaningful activity and enhancing recovery, as defined using connectedness, hope, identity, meaning and empowerment (CHIME) principles. An eight -session PPIE programme (“Discovery Group”) was designed to support patient-led research in a secure mental health hospital. This feasibility study aims to evaluate the acceptability of the programme from the perspective of patients and identify potential outcomes.

**Methods:**

A retrospective single-arm post-programme evaluation of Discovery Group was undertaken. Participants attended an evaluation workshop where they were interviewed individually to complete an acceptability questionnaire designed using the domains of the Theoretical Framework of Acceptability. Participants also completed an outcomes questionnaire, which included CHIME-based recovery items. Quantitative data were analysed descriptively. Direct content analysis was applied to qualitative data.

**Results:**

In our sample, eight participants attended at least one session of the discovery group with one patient attending all sessions. Most of the participants felt positive about taking part in the group and expressed interest in joining another group in future. All participants experienced some burden from the effort required during group sessions, but a low level of opportunity cost in terms of the extent to which they perceived they had to forfeit benefits to participate in the programme.Some described the group as effective in helping them learn about research. Of the five CHIME recovery domains, only connectedness was reported as a benefit of the group. The participants valued the opportunity to use their time.

**Conclusion:**

Discovery Group is a novel intervention that offers high level, non-tokenistic PPIE suitable for use in secure mental health inpatient settings. It produces research of value to patients through a programme of high acceptability and provides them with potential benefits of recovery as well as research knowledge and skills, and an activity that alleviates boredom, enhances autonomy, breaks down some important power and paternalistic barriers that can be experienced by patients detained in secure mental health settings. Finally, a future evaluation study that involves patients during the design, implementation, evaluation and writing stages, aiming to measure the potential outcomes identified in the present study using pre- and post-testing with a control group would reliably demonstrate the effectiveness of the revised Discovery Group and ensure meaningful involvement with patients as co-researchers.

